# YOLO-LF: application of multi-scale information fusion and small target detection in agricultural disease detection

**DOI:** 10.3389/fpls.2025.1609284

**Published:** 2025-09-11

**Authors:** Xinming Wang, Sai Hong Tang, Mohd Khairol Anuar B. Mohd Ariffin, Mohd Idris Shah B. Ismail, Jiazheng Shen

**Affiliations:** Faculty of Engineering, Universiti Putra Malaysia, Serdang, Malaysia

**Keywords:** YOLO-LF, small target detection, agricultural disease detection, multi-scale information fusion, deep learning, YOLOv11

## Abstract

With the increasing threat of agricultural diseases to crop production, traditional manual detection methods are inefficient and highly susceptible to environmental factors, making an efficient and automated disease detection method urgently needed. Existing deep learning models still face challenges in detecting small targets and recognizing multi-scale lesions in complex backgrounds, particularly in terms of multi-feature fusion. To address these issues, this paper proposes an improved YOLO-LF model by introducing modules such as CSPPA (Cross-Stage Partial with Pyramid Attention), SEA (SeaFormer Attention), and LGCK (Local Gaussian Convolution Kernel), aiming to improve the accuracy and efficiency of small target disease detection. Specifically, the CSPPA module enhances multi-scale feature fusion, the SEA module strengthens the attention mechanism for contextual and local information to improve detection accuracy, and the LGCK module increases the model’s sensitivity to small lesion areas. Experimental results show that the proposed YOLO-LF model achieves significant performance improvements on the Plant Pathology 2020 - FGVC7 and Plant Pathology 2021 - FGVC8 datasets, particularly in mAP@0.5% and mAP@0.5-0.95%, outperforming existing mainstream models. These results indicate that the proposed method effectively handles complex backgrounds and small target detection tasks in agricultural disease detection, demonstrating high practical value.

## 1 Introduction

Agriculture is the foundational industry for national economic and social development. The high incidence and spread of crop diseases pose a serious threat to food security, agricultural product quality, and the stability of agricultural ecosystems Ashok et al. (2020); Harakannanavar et al. (2022). According to statistics from the United Nations Food and Agriculture Organization (FAO), crop yield losses due to pests and diseases account for 20% to 40% of global production annually, resulting in direct economic losses of hundreds of billions of dollars. Traditional crop disease identification relies on the experience of agricultural experts, which is plagued by high labor costs, low efficiency, and subjectivity, making it difficult to meet the demands of modern, intelligent, and precise agriculture. Therefore, the development of efficient and automated crop disease detection technologies has become an important research direction in the field of smart agriculture Eunice et al. (2022); Vasavi et al. (2022).

In recent years, with the widespread application of deep learning in the field of computer vision, disease detection methods based on Convolutional Neural Networks (CNNs) have demonstrated strong feature extraction and image recognition capabilities, providing new approaches for intelligent crop disease detection Kundu et al. (2022); Chowdhury et al. (2021). Related research has gradually expanded from early image classification tasks to more refined tasks such as object detection and instance segmentation, enabling precise localization and identification of disease spots. Among them, the YOLO (You Only Look Once) series, as an end-to-end single-stage object detection algorithm, has become one of the mainstream choices for disease detection tasks in agricultural scenarios due to its fast detection speed, lightweight structure, and flexible deployment advantages Zhao et al. (2021).

Deep learning methods for plant leaf disease detection primarily use Transformer and CNN architectures, which have made significant progress in small object detection tasks Li et al. (2025); Zhang et al. (2025). Transformer architectures, particularly the Conditional Generative Adversarial Network (C-GAN) Abbas et al. (2021), have been widely applied in tomato disease detection. Through transfer learning on DenseNet121, experimental results show that on the PlantVillage dataset, the accuracy rates for five, seven, and ten categories are 99.51%, 98.65%, and 97.11%, respectively Lin et al. (2022). In object detection, models combining unsupervised training and transfer learning have built diagnostic models through the upward network and DETR (Detection Transformer), achieving a recognition rate of 96.2% for citrus Huanglongbing Lin et al. (2022). Additionally, the Swin Transformer uses a hierarchical window attention mechanism, offering significant advantages in multi-scale feature capturing, especially in agricultural images with complex backgrounds and noise Khattak et al. (2021). It performs exceptionally well in detecting small lesion areas. The Vision Transformer (ViT) divides images into fixed size patches and uses the self-attention mechanism to capture global information, effectively handling small lesion areas, especially when the background is complex and heavily occluded. Deformable DETR, an improvement of the traditional DETR model, introduces deformable convolution and selective attention mechanisms to enhance detection accuracy in small lesion areas. By dynamically adjusting the receptive field, Deformable DETR can better handle targets of varying sizes, particularly in agricultural disease detection, where it accurately identifies small lesion areas Yang et al. (2023); Zhu et al. (2020).

In CNN architectures, the YOLO series models have become mainstream methods for agricultural disease detection due to their efficient real-time inference capabilities. A lightweight model based on YOLOv5, optimized through its backbone network and detection head, successfully achieves efficient recognition of various crop diseases and achieves significant detection accuracy on large public datasets Mathew and Mahesh (2022). These improvements allow YOLOv5 to maintain high accuracy even under resource-limited conditions, making it especially suitable for disease monitoring on mobile and embedded devices Mahum et al. (2023). To meet the real-time and deployment convenience needs of agricultural scenarios, researchers have further optimized the YOLO architecture by introducing efficient network modules. For example, lightweight convolution networks like GhostNet and ShuffleNet have been integrated into the YOLO backbone, effectively reducing computation and memory consumption, adapting to real-time detection requirements on mobile devices and edge computing platforms Tiwari et al. (2020). This optimization enables YOLO to run efficiently on small hardware platforms, providing practical solutions for large-scale agricultural disease monitoring. Moreover, to improve the model’s adaptability to complex backgrounds, the YOLO series has incorporated various attention mechanisms to enhance the model’s sensitivity to critical information. For example, the Squeeze-and-Excitation (SE) module adjusts channel feature weights adaptively, focusing on the lesion areas, thereby improving detection accuracy Panigrahi et al. (2020). In more complex backgrounds, attention modules such as CBAM (Convolutional Block Attention Module) combine spatial and channel attention, enhancing the model’s adaptability in challenging environments with high noise, lighting variations, and object occlusion Islam et al. (2021).

Nevertheless, the existing YOLO series models still face certain challenges in crop disease detection. First, the feature extraction structure is insufficient in responding to small-scale lesions. Traditional convolutional networks have limitations in multi-scale information fusion, which results in small targets, such as disease spots, being weakened during downsampling, thereby affecting the overall detection accuracy. Second, the design of attention mechanisms is relatively simplistic and struggles to handle the complex field environments. Existing methods often focus on enhancing information in either the channel or spatial dimension, lacking the joint modeling of cross-scale and local spatial information, which limits their robustness.

To address this, this paper proposes an improved lightweight disease detection model, YOLO-LF, based on YOLOv11 Khanam and Hussain (2024). Specifically, YOLO-LF maintains the high-speed detection advantages of the YOLO series while optimizing the feature extraction structure and attention mechanisms to improve the model’s detection accuracy and generalization ability in complex agricultural environments. On one hand, this paper introduces the CSPPA (Cross-Stage Partial with Pyramid Attention) structure. This structure enhances the representation ability of different scale disease spots in the backbone network with low computational overhead, effectively mitigating the issue of small targets being overlooked and improving the semantic integrity and detail retention of the model. On the other hand, this paper introduces the SEA (Spatial-Enhanced Attention) mechanism, which jointly models spatial and channel information during feature fusion. The SEA module guides the model to focus on the salient features of the disease spot region by constructing spatial dependency perception paths and a global channel weighting strategy, suppressing background interference and further enhancing the robustness and accuracy of disease detection.

The contributions of this paper are as follows:

- This paper proposes an improved version of YOLOv11, called YOLO-LF (YOLO with Lightweight Fusion), which enhances small target detection accuracy and model efficiency in agricultural disease detection tasks by introducing modules such as CSPPA, SEA, and LGCK. This approach addresses the challenges of detecting small lesions in complex backgrounds, a common issue with traditional methods.
- The paper introduces the CSPPA module, which enhances multi-scale information fusion and optimizes the model’s attention mechanism, improving detection performance in complex scenarios.
- Experiments on the Plant Pathology 2020 - FGVC7 and Plant Pathology 2021 - FGVC8 datasets show that the proposed model significantly outperforms existing mainstream models in several metrics, demonstrating the effectiveness and practical application potential of the method in agricultural disease detection.

## 2 Related work

### 2.1 The research on plant disease detection based on CNN

In recent years, convolutional neural network (CNN)-based plant disease detection technology has made significant progress in improving model accuracy and detection efficiency He et al. (2025). First, some studies have combined deep convolutional neural networks (DCNN) with data augmentation techniques to enhance the model’s robustness and generalization ability. By introducing data augmentation methods such as image rotation and scaling, these approaches significantly improve performance on small sample datasets. However, despite enhancing recognition accuracy, the model’s performance is still lacking when faced with high noise and complex backgrounds, and its adaptability to different plant species is poor Agarwal et al. (2020); Xing et al. (2025) Next, some studies have employed multitask learning (MTL) methods to combine plant disease classification and localization tasks to further improve detection performance. This approach shares feature extraction layers across tasks, allowing the network to learn more general features, thus improving detection accuracy and training efficiency. However, these methods require significant computational resources, and there are certain bottlenecks when processing large-scale data Sun et al. (2020). Additionally, to address small lesion detection, some studies have proposed multi-scale convolutional neural networks (MSCNN) to extract features at different scales, thereby improving the ability to detect small lesions. These methods are effective at handling lesions of various sizes, especially in complex backgrounds and low-contrast images. However, the computational cost of the multi-scale feature extraction process is high, leading to challenges in real-time performance Lauguico et al. (2020). As research progressed, some scholars combined convolutional neural networks with image segmentation techniques to propose joint detection and segmentation models. These methods accurately segment lesion regions and combine deep learning models for disease recognition, achieving good results. Although these methods improve lesion localization precision, the computational complexity of the image segmentation process and long model training times limit their practical application Kaushik et al. (2020); Babulal et al. (2022). In recent years, some studies have introduced self-attention mechanisms (such as SE-Net and Transformer) to strengthen the network’s focus on key features. With this mechanism, the model can pay more attention to lesion regions, improving detection accuracy. These methods have shown improved performance in complex backgrounds, but they still face high computational complexity issues, particularly when real-time processing is required on resource-constrained devices Masenya (2024). Moreover, recent studies have proposed a model based on generative adversarial networks (GANs) to generate high-quality synthetic images to enhance the diversity of training data. These methods effectively solve the problem of insufficient data, but due to the gap between generated and real images, they may affect the model’s generalization ability, especially in scenes with special backgrounds and lighting conditions, where detection performance may decline Shin et al. (2021). To further improve small target detection in agricultural disease detection, some methods have drawn inspiration from advances in medical image segmentation techniques, such as the Context Aware Network with Dual-Stream Pyramid (CANet) Xie et al. (2023). This method, originally designed for medical image segmentation, addresses challenges similar to those encountered in plant disease detection, such as varying object scales, complex backgrounds, and similar appearances between objects. CANet includes a dual-stream pyramid module and an encoder-decoder module with context awareness. The dual-stream pyramid captures complementary features at different layers by using multi-resolution input versions and multi-scale convolutional units, which is particularly helpful for learning small lesion features at various scales. In addition, PIF-Net Xie et al. (2025), through the design of parallel paths, enables the network to process different features at multiple scales simultaneously, and strengthens the connections between layers through an interaction fusion mechanism, thereby improving the detection capability for diverse plant diseases. This network not only effectively extracts local details of plant lesions but also maintains high detection accuracy in complex backgrounds. Particularly when facing small lesion areas, it can accurately segment and identify lesion features, thus enhancing the overall performance of agricultural disease detection.

Overall, CNN-based plant disease detection methods have made progress in terms of accuracy, robustness, and real-time performance. However, issues such as computational complexity, data diversity, and adaptability to different plant species remain, and how to ensure high accuracy while improving model real-time performance and adaptability remains a major challenge in current research.

### 2.2 The research on plant disease detection based on YOLO

The plant disease detection research based on the YOLO (You Only Look Once) architecture has evolved through several versions, from YOLOv5 to YOLOv11, with each version gradually improving the accuracy and efficiency of disease detection in the agricultural field. YOLOv5 Ma et al. (2023) introduced CSPNet and cross-stage feature fusion, enhancing detection accuracy and model compression capabilities. Its high detection speed and small model size made it suitable for mobile devices and real-time monitoring, but its accuracy in detecting small lesions in complex backgrounds still needs improvement. YOLOv6 Li et al. (2022) adopted PANet and CSPDarkNet53, which enhanced multi-scale detection capabilities. Through multi-scale support and strengthened loss functions, it performed better in detecting small lesions, but still faced issues with occlusion and lighting changes in complex backgrounds. YOLOv7 Wang et al. (2023a) introduced EfficientRep and efficient convolutions, optimizing computational efficiency and detection accuracy. By using convolution redirection and data augmentation techniques, it improved the model’s performance in multi-scale object detection, but its adaptability to complex scenes remained limited. YOLOv8 Liu et al. (2023) introduced efficient integration techniques and lightweight convolutions, improving overall efficiency. By enabling fast parameter tuning and structural optimization, it accelerated the training process, but its computational cost remained high, especially on resource-limited devices, where real-time performance was still a challenge. YOLOv9 Wang et al. (2024d) further optimized the model, using adaptive convolutions and new loss functions to improve the accuracy of small lesion detection. Its feature fusion and multi-scale convolution enhancement made it more adaptable to complex agricultural environments, but it still consumed considerable computational resources. YOLOv10 Wang et al. (2024b) introduced a multitask convolution model, improving multi-task handling capabilities. Through detail optimization and multi-scale data fusion, it enhanced the model’s performance, but it still required a large amount of computational resources for training, especially on large-scale datasets. YOLOv11 Khanam and Hussain (2024), building upon YOLOv10, introduced CSPPA-enhanced feature maps, significantly improving disease detection accuracy. It combined deep learning with network optimizers, and although it required substantial computational resources when training large-scale datasets, its ability to detect small lesions and adapt to complex backgrounds was significantly enhanced.

The YOLO-LF model proposed in this paper further improves the accuracy and efficiency of small lesion detection by introducing modules such as CSPPA, SEA, and LGCK. The CSPPA module enhances multi-scale feature fusion, the SEA module strengthens the attention mechanism for contextual and local information, and the LGCK module increases the model’s sensitivity to small lesion areas. The improvements to YOLO are specifically shown in **Table 1**.

**Table 1:** YOLO versions: innovations, training strategies, and optimization techniques.

YOLO version	Innovation	Training strategy	Optimization technique
YOLOv5	CSPNet, cross-stage feature fusion, enhanced model compression ability	Mosaic, Cumulix, data augmentation, detection capability correction	Object detection, classification loss, gradient loss
YOLOv6	PANet, CSPDarkNet53	Strengthened loss functions, stronger multi-scale support	Efficient data augmentation, image data enhancement
YOLOv7	EfficientRep, introduced efficient convolutions	Activated networks, convolution redirection, data augmentation	Fast gradient descent, real-time efficient enhancement
YOLOv8	Efficient integration techniques and lightweight convolutions	Structural optimization, fast parameter tuning	Efficient convolutions, batch normalization
YOLOv9	Efficient convolutions and new loss functions	Feature fusion, multi-scale convolution enhancement	Adaptive optimization, custom normalization layers
YOLOv10	Efficient multi-task convolution model	Network integration, detail optimization, efficient training	Detail multi-scale data fusion, adaptive training process
YOLOv11	Introduced CSPPA enhanced feature maps based on YOLOv10	Combined deep learning with network optimizers	Accelerated network deployment, reduced resource consumption, and training errors
YOLO-LF	CSPPA, SEA, LGCK modules for small lesion detection	End-to-end training, multi-scale and small target detection	Accelerated network deployment, reduced resource consumption, and training errors

## 3 Method

### 3.1 Overall network architecture

**Figure 1:**
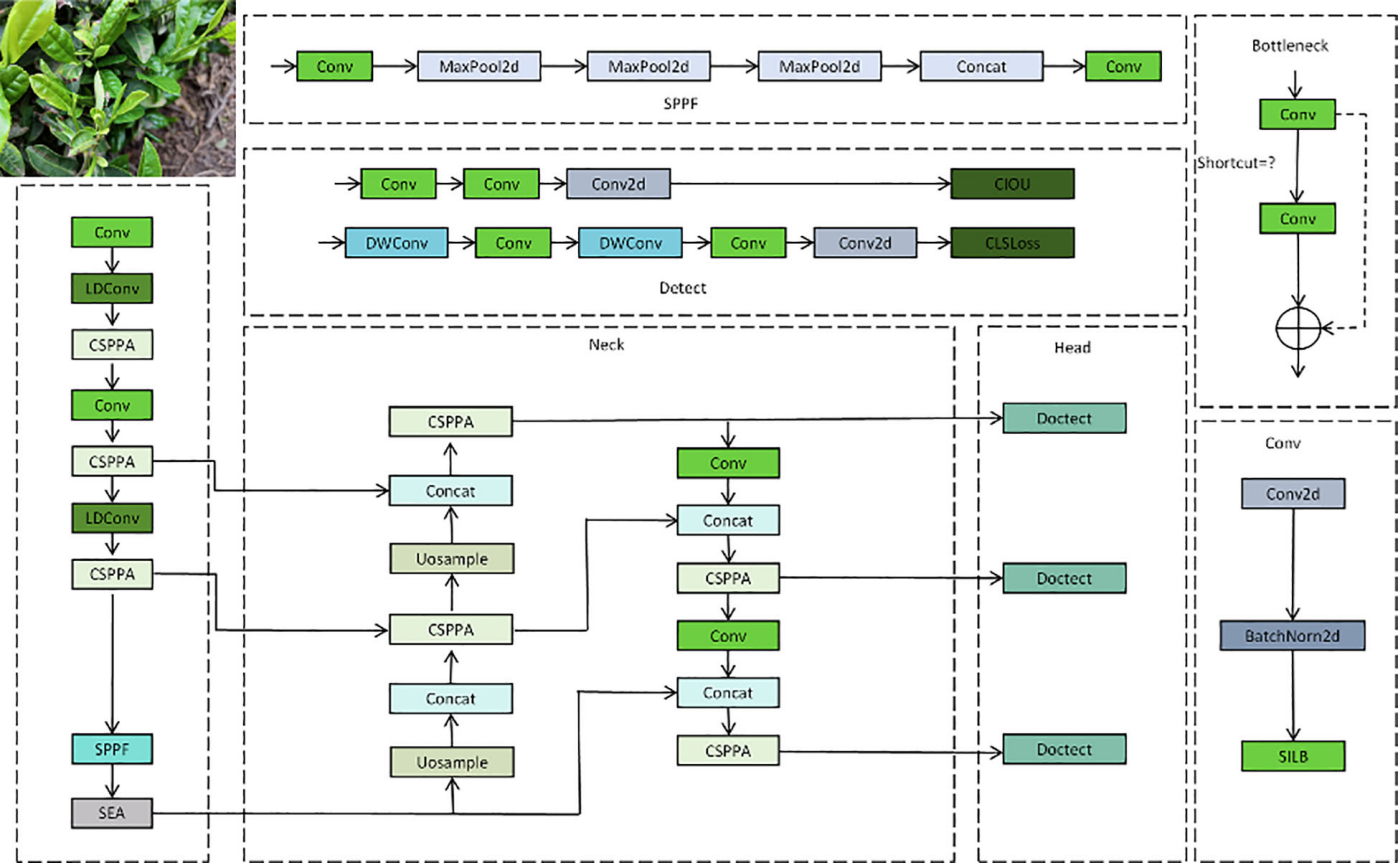
The overall network architecture of YOLO-LF, including the backbone, neck, and head modules.

This paper proposes an improved version of YOLO-LF (YOLO with Lightweight Fusion), which enhances YOLOv11 to improve the precision and efficiency in agricultural disease detection tasks. The network architecture of this model is mainly divided into three parts: Backbone, Neck, and Head. These modules work together for efficient feature extraction, fusion, and detection, enabling the model to effectively identify lesions and other targets in complex backgrounds. As shown in **Figure 1**, the overall architecture of the network can be divided into several key modules, each playing a crucial role in enhancing the performance of the model.

In the Backbone part, YOLO-LF first processes the input image through multiple convolution layers. The primary task of this part is to extract basic features from the image. The first part of the diagram includes a series of convolution (Conv) and pooling (MaxPool2d) operations, gradually reducing the spatial dimensions of the feature map while strengthening the capture of key image features. Additionally, the CSPPA module is used in the Backbone to enhance the flow of information across stages and the fusion of multi-scale features, which is especially important for detecting small-scale lesions. Furthermore, the LDConv (Local Convolution) module is utilized to extract local features, enhancing sensitivity to small lesion areas. The SPPF (Spatial Pyramid Pooling Fusion) module is employed to introduce multi-scale information into the feature map, allowing the model to handle targets of various sizes, which is critical for detecting small lesions in complex backgrounds. The Neck part is responsible for effectively fusing the multi-scale features extracted by the Backbone and providing more precise feature representations for the Head. In the Neck module, multiple CSPPA layers are stacked, gradually increasing the resolution of the feature map to better process detail information. The Uosample (Upsampling) operation is also used in the Neck to recover the spatial dimensions of the feature map through upsampling, ensuring that detail information is not lost. The Concat operation is used to merge feature maps from different levels, making full use of multi-layer feature information. The final output of this part is further processed by convolution layers, providing rich and precise features for subsequent object detection. The Head part is responsible for performing object detection based on the feature map output by the Neck. Specifically, the Head module uses the Doctect operation to identify the target regions and output the final prediction results. The model employs CIOU (Complete Intersection over Union) as the loss function to help the network more accurately regress the coordinates of the target bounding box. Meanwhile, CLS Loss (Classification Loss) is used to optimize the class prediction, ensuring that the model not only accurately locates the targets but also classifies them correctly. It is worth noting that YOLO-LF further optimizes the network by introducing the SEA (Sea Attention) module, which helps the model better capture both the contextual and local details in the image. This enhances the model’s attention ability, especially in complex scenes.

By improving YOLOv11, the YOLO-LF model introduces various innovative modules, such as CSPPA, LDConv, SPPF, SEA, etc., which significantly enhance the model’s ability to detect agricultural diseases. The entire network architecture reasonably distributes the tasks of Backbone, Neck, and Head, enabling the model to efficiently perform feature extraction, fusion, and object detection, particularly for multi-scale and multi-feature lesion detection tasks in complex backgrounds.

### 3.2 Cross-stage partial with pyramid attention

The CSPPA structure is a deep learning architecture designed to enhance the multi-scale information fusion capability in image processing, particularly suitable for agricultural disease detection tasks. This structure addresses challenges in traditional convolutional neural networks, such as information loss and excessive computational overhead, by introducing cross-stage partial connections and pyramid attention mechanisms. The core idea is to reduce redundant computations through cross-stage partial connections and dynamically weight information from different scales using the pyramid attention mechanism, thus improving the model’s robustness and accuracy in complex environments.

**Figure 2:**
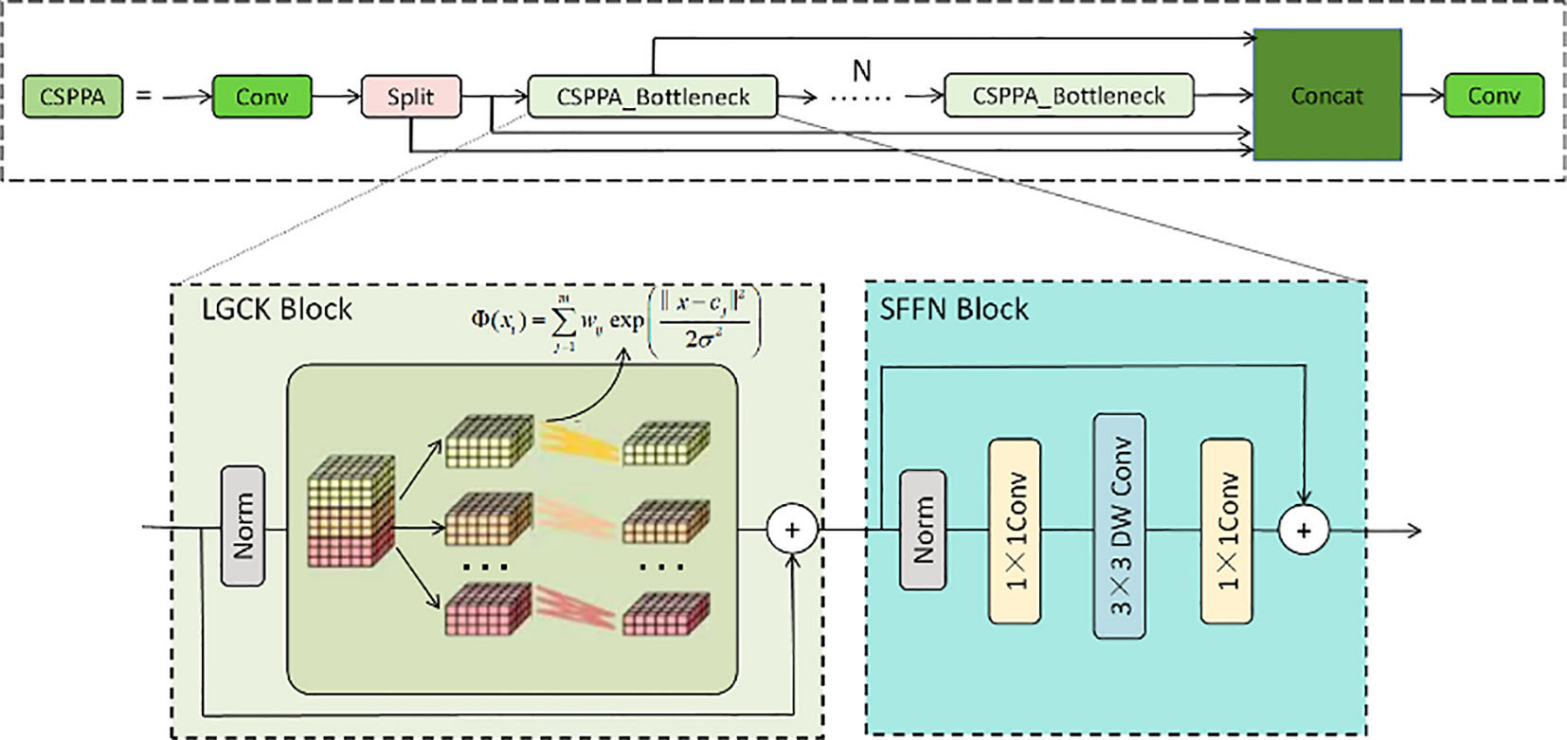
The CAPPA network structure, including the CSPPA module, LGCK Block (Local Gaussian Convolution Kernel), and SFFN Block (Spatial Feature Fusion Network), designed to enhance multi-scale information fusion and small target detection in agricultural disease detection tasks.


As shown in **Figure 2**, the main component of the CSPPA structure is the CSPPA Bottleneck, which consists of two key modules: the LGCK (Local Gaussian Convolution Kernel) Block and the SFFN (Spatial Feature Fusion Network) Block. The LGCK Block enhances the model’s response to small-scale lesions by applying a local convolution kernel and a weighted fusion strategy. Specifically, the LGCK Block uses a Gaussian-based weighting mechanism that adjusts the convolution kernel’s center and standard deviation to process the input features. The core formula of this mechanism is as follows:

$$\text{Φ}(x)=\sum_{j=1}^{m}w_{j}\exp\;(\frac{∥x-c_{j}{∥}^{2}}{2\sigma^{2}})$$

where *x* represents the input feature map, *c_j_* is the center of the *j*-th convolution kernel, *σ* is the standard deviation of the Gaussian function, *w_j_* is the weight of the *j*-th convolution kernel, and *m* is the number of convolution kernels.

To further enhance the performance of the LGCK Block, we introduce a dynamic adjustment mechanism, where *σ* (the standard deviation of the Gaussian kernel) dynamically varies based on the input features. This adjustment mechanism can be expressed by the following formula:


$$\sigma (x)=\sigma_{0}\cdot (1+\lambda \cdot \frac{\text{Var}(x)}{\text{Max}(\text{Var}(x))})$$

where *σ*_0_ is the initial standard deviation, *λ* is the adjustment coefficient, Var(*x*) is the local variance of the input feature map *x*, and Max(Var(*x*)) is the maximum variance of all feature maps. Through this dynamic adjustment, the receptive field of the convolution kernel changes based on the local features of the input, enhancing the model’s adaptability to small lesion areas, especially when the target regions are small or the background is complex.

The SFFN Block further enhances the feature representation ability through a series of convolution operations. It uses a combination of 1×1 convolution and 3×3 depthwise convolution. First, a 1×1 convolution is applied to reduce the feature map’s dimensionality and perform channel compression, followed by a 3×3 depthwise convolution to extract spatial features. Finally, a 1×1 convolution is used for feature fusion to ensure that features from different layers are effectively combined. This design not only enhances the model’s ability to capture local spatial information but also reduces computational overhead, improving the model’s deployment efficiency on mobile devices. The computation process of the SFFN Block can be described as:


Output=Norm(1×1Conv(DWConv(3×3)))

In the CSPPA model, the introduction of the pyramid attention mechanism enables the model to adaptively weight features from different scales, thereby improving the fusion of multi-scale information. The mechanism works by assigning a learned weight coefficient to each scale feature map to determine the importance of features at each scale. The weighting process of the pyramid attention mechanism is as follows:


$$A(x)=\sum_{i=1}^{N}\alpha_{i}\cdot F_{i}(x)$$

where *A*(*x*) represents the final weighted output, *F_i_* (*x*) is the feature map at the *i*-th scale, and *α_i_* is the weight coefficient for the *i*-th scale. This mechanism allows the CSPPA model to effectively extract and fuse information at different scales, especially when faced with complex backgrounds and noisy disturbances, providing more accurate detection results.

Through these innovative designs, the CSPPA model optimizes the performance of traditional convolutional neural networks in small target detection, particularly for agricultural disease detection. The advantage of this structure lies in its ability to significantly improve detection accuracy while reducing computational costs, especially in multi-scale information fusion and complex background handling. By combining cross-stage partial connections and pyramid attention mechanisms, the CSPPA model not only enhances the response to small targets but also improves overall detection robustness, making it a valuable tool for large-scale agricultural monitoring applications.

### 3.3 SEA module

**Figure 3:**
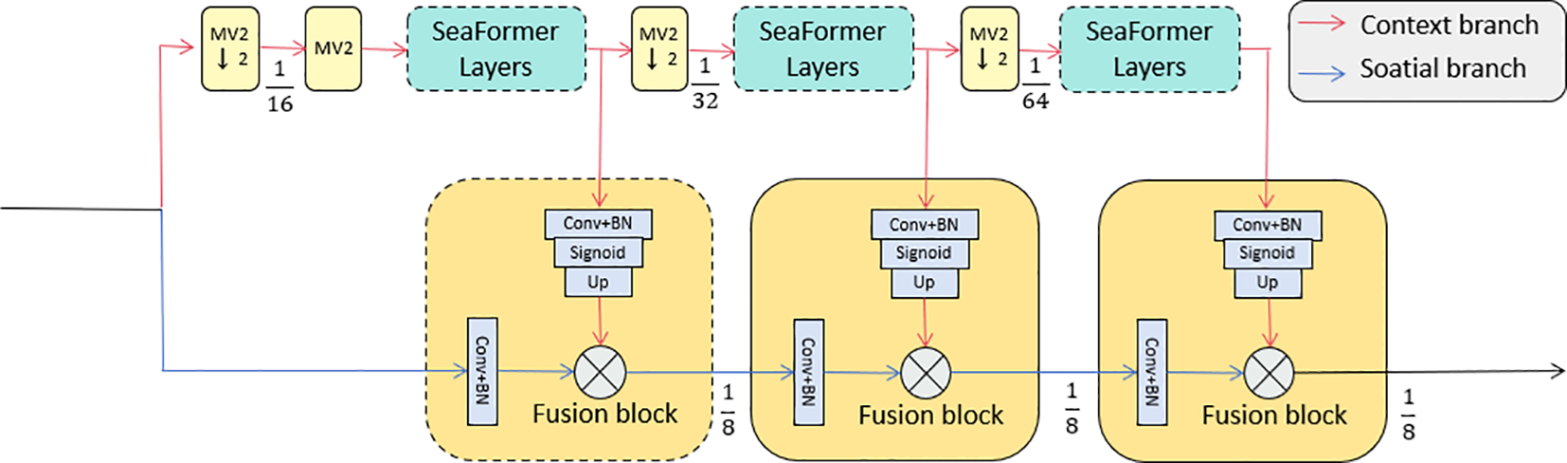
The SEA attention mechanism network structure, including the context branch and spatial branch, with fusion blocks designed to effectively capture global and local dependencies in agricultural disease detection tasks.

The SEA attention (SeaFormer Attention) mechanism is a core component of the SeaFormer architecture (As shown in **Figure 3**) Wan et al. (2023). It handles global and local information through the context branch and the spatial branch, respectively. This design allows the model to capture multidimensional dependencies in complex tasks such as agricultural disease detection, thereby enhancing the model’s ability to identify important features. In such tasks, effectively combining global context and local details is crucial for improving accuracy.

In the SeaFormer architecture, the input feature map is processed by two branches: the context branch and the spatial branch. The context branch is designed to capture long-range dependencies in the image, focusing on global contextual information, while the spatial branch is focused on capturing fine-grained local details. This way, the model can form an effective complement between global and local information, thus improving detection accuracy.

Specifically, the input feature map is represented as *F* ∈ ℝ*^H^* ^×^ *^W^* ^×^ *^C^*, where *H* is the image height, *W* is the width, and *C* is the number of channels. In the context branch, the input features are projected through linear transformations to generate query (*Q_c_*), key (*K_c_*), and value (*V_c_*) matrices. Then, the context attention map *A_c_* is computed by taking the inner product between the query matrix and the key matrix to capture global dependencies. The calculation formula is as follows:


$$Q_{c}=F\cdot W_{q}+b_{q}$$

$$K_{c}=F\cdot W_{k}+b_{k}$$

$$V_{c}=F\cdot W_{v}+b_{v}$$

where *W_q_*, *W_k_*, and *W_v_* are the projection matrices for the query, key, and value, and *b_q_*, *b_k_*, and *b_v_* are the corresponding bias terms. The context attention map *A_c_* is calculated as follows:


$$A_{c}=\text{softmax }(\frac{Q_{c}\cdot K_{c}^{T}}{\sqrt{d_{k}}})$$

where *d_k_* is the dimensionality of the key vector, and $K_{c}^{T}$ is the transpose of the key matrix.

Next, in the spatial branch, the feature map *F* is processed through a convolution operation to capture local spatial information. Specifically, the spatial attention map *A_s_* is computed via the convolution operation:


$$A_{s}=\text{Conv}2\text{D}(F,W_{s})$$

where *W_s_* is the convolution kernel. The resulting spatial attention map *A_s_* is normalized to emphasize the most relevant spatial regions in the image:


$$A_{s}=\frac{A_{s}}{\sum A_{s}}$$

Subsequently, the outputs of the context and spatial branches are combined through a fusion module to obtain a refined feature map. During the fusion process, the context and spatial attention maps are element-wise multiplied and then further processed through a convolution operation to obtain the final fused feature map *F*_fusion_:


$$F_{\text{fusion}}=\text{Conv}2\text{D }(\text{Sigmoid}(A_{c}\times A_{s}),W_{f})$$

where *A_c_* ×*A_s_* is the element-wise multiplication of the context and spatial attention maps, and *W_f_* is the learnable weight for the fusion operation.

In the attention mechanism computation, matrix multiplication and attention calculation are often used to compute the similarity between queries and keys. For example, the inner product between the query matrix *Q* and the key matrix *K* yields an attention score matrix. To ensure the normalization of the attention weights, the softmax function is typically applied as follows:


$$\text{Softmax }(x_{i})=\frac{e^{x_{i}}}{\sum_{j}e^{x_{j}}}$$

Additionally, in certain attention mechanisms, especially in spatially aware architectures, integration operations are used to capture global dependencies. This can be represented as:


$$\int_{R}A(x,y)\;dx\;dy$$

where *R* is the region of interest, and *A*(*x,y*) is the attention map within this region.

Finally, after fusion and refinement, the feature map undergoes an upsampling operation to restore it to the original resolution:


$$F_{\text{upsampled}}=\text{UpSampling}(F_{\text{fusion}},\text{scale})$$

In summary, the SEA attention mechanism combines the context and spatial branches to allow the SeaFormer model to efficiently capture global and local details in images. In complex tasks like agricultural disease detection, considering both global context and local spatial information is crucial, and this multi-dimensional feature fusion significantly enhances the model’s performance. With this design, SeaFormer not only strengthens the model’s attention capability but also effectively handles the complexities of spatial and contextual information, providing more precise feature representations for detection tasks.

## 4 Experiments

### 4.1 Datasets

The datasets used in this paper include Plant Pathology 2020 - FGVC7 Thapa et al. (2020) and Plant Pathology 2021 - FGVC8 (Dataset Fruit Pathology, S, 2021), both of which are from the FGVC (Fine-Grained Visual Categorization) challenge in the field of plant pathology. These datasets are specifically designed for image classification tasks related to plant diseases, covering a variety of plant disease lesions and providing rich annotated data, aimed at advancing the development of plant disease detection technology.

The Plant Pathology 2020 - FGVC7 dataset includes lesion images from various plants, and we selected annotations for 4 different types of plant diseases. With a large amount of data, it effectively supports the training and validation of deep learning models. The dataset offers precise annotations of lesion types and locations, providing researchers with rich diversity and high-quality training data.

The Plant Pathology 2021 - FGVC8 dataset is an upgraded version of the FGVC series, further expanding the number of disease categories and images, especially strengthening the handling of disease diversity across different plant species and environmental conditions. This dataset not only adds more image samples but also optimizes disease category classification and image quality, further enhancing the recognition ability for the diverse manifestations of plant diseases.

**Figure 4:**
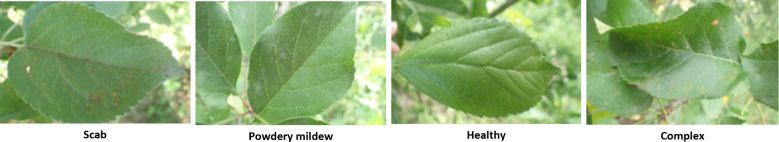
The dataset example showcases different leaf conditions: scab, powdery mildew, healthy, and complex.

In this study, the Plant Pathology 2020 - FGVC7 and Plant Pathology 2021 - FGVC8 datasets were divided into training, validation, and test sets. The training set accounts for 70% of the dataset and is used for model training and parameter tuning; the validation set accounts for 15% and is used to evaluate the model’s performance and fine-tune hyperparameters during training; the test set accounts for 15% and is used for the final evaluation of the model’s performance on unseen data. A sample of the dataset is shown in **Figure 4**.

### 4.2 Experimental details

**Figure 5:**
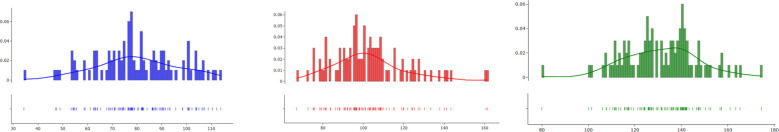
The color channel distribution (blue, red, and green) is shown.

Data Preprocessing In the data preprocessing stage, we first analyze the RGB channels of the dataset. **Figure 5** shows the information from the RGB channels of the dataset. It can be observed that there are certain differences in the color distribution across different lesion types and plant species, which are crucial for disease recognition. To enhance the model’s generalization ability and improve training performance, we applied data augmentation techniques.

**Figure 6:**
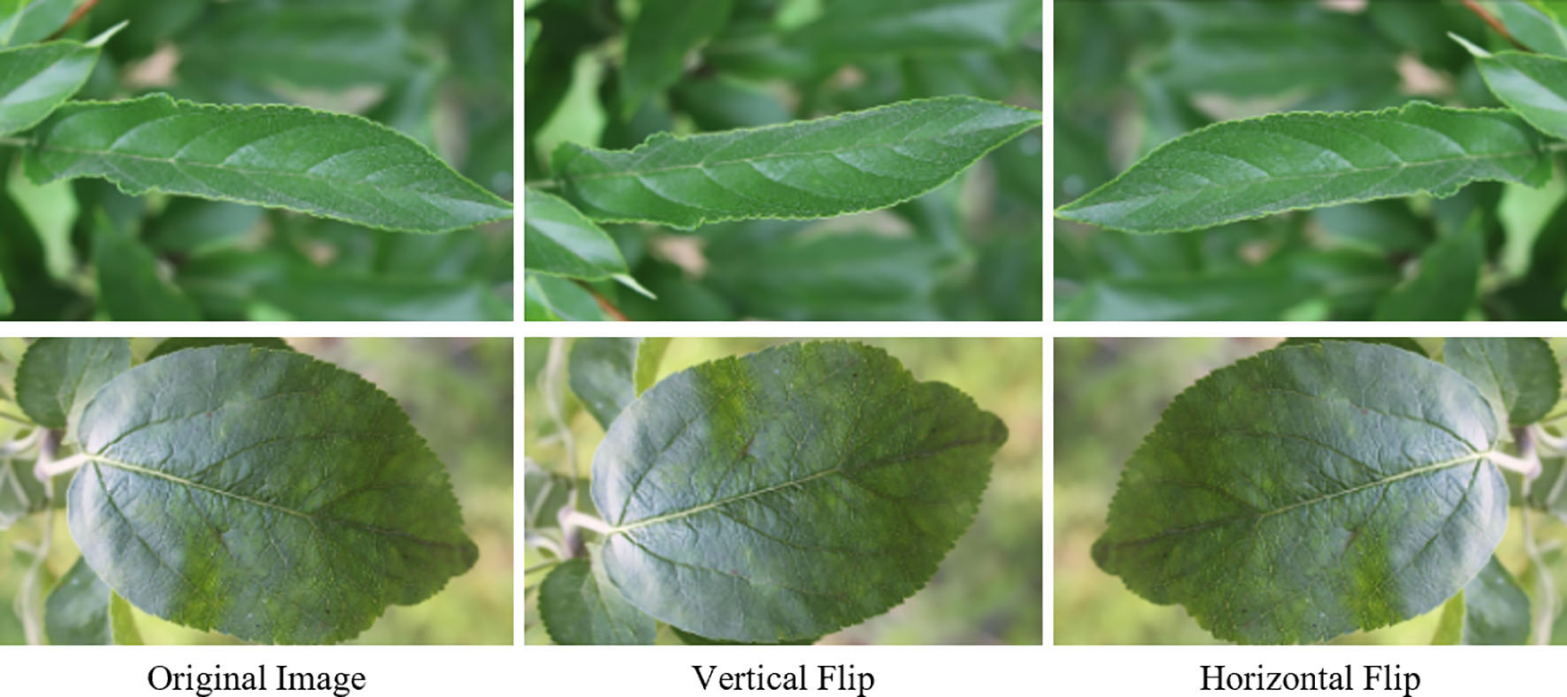
Data augmentation techniques are demonstrated on leaf images, showing the original image, vertical flip, and horizontal flip.

To increase the training samples and reduce the risk of overfitting, we employed flip transformations. Specifically, images in the dataset were augmented by performing horizontal and vertical flips. **Figure 6** shows the images in the dataset after flip augmentation. The flip operation effectively increased the diversity of the samples, allowing the model to train on lesions in various orientations, thus improving the model’s adaptability to different diseases.

**Figure 7:**
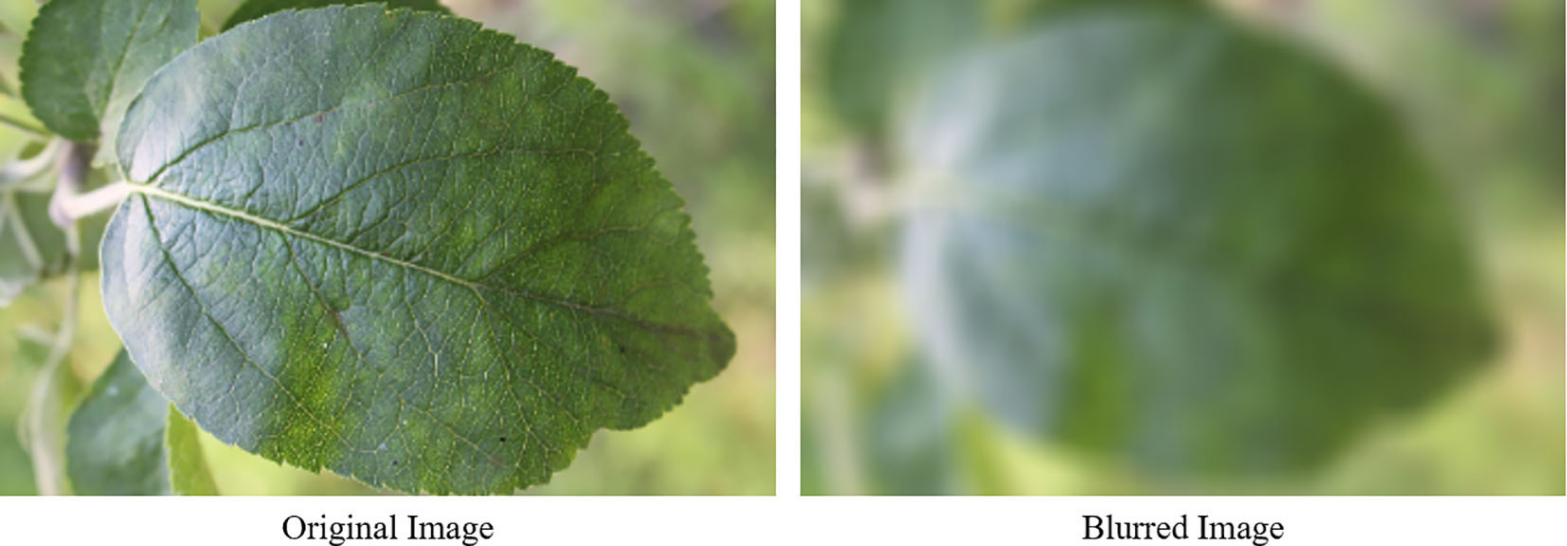
Illustration of image blurring as a data augmentation technique for leaf disease detection.

To enhance the image robustness and handle complex backgrounds, we applied blur processing to the dataset. **Figure 7** shows the effect of applying blur processing to the dataset images. The blur processing simulates image blurring under different shooting conditions, helping the model better adapt to real-world low-quality or blurry images. This processing not only improved the model’s stability in handling real-world scenarios but also enabled the model to better recognize lesion regions, even when the image quality decreased.

The input image size of the YOLO-LF model is 256 × 256 × 3. After convolution and pooling operations in the Backbone section, the feature map gradually reduces to 64 × 64 × 128. On this basis, the CSPPA and LDConv modules enhance feature extraction, while the SPPF module fuses multi-scale information, resulting in a final output feature map of 64 × 64 × 128. In the Neck section, upsampling and concatenation operations are used to fuse feature maps of different scales, with the output size being 128 × 128 × 256, which is then passed to the Head module for object detection.

#### 4.2.1 Experimental environment

In terms of hardware, the experiment was conducted on a computer equipped with an NVIDIA GeForce RTX 3090 graphics card, which is a high-performance GPU capable of effectively accelerating the training and inference processes of deep learning models. The system also features an Intel Core i9-10900K processor with 10 cores and 20 threads, providing high-performance computing power to ensure efficient execution of data preprocessing and model training tasks. Additionally, the system is configured with 64GB DDR4 memory and 1TB SSD storage, ensuring the capability to handle large-scale datasets and meet the requirements for fast read and write operations.

In terms of the software environment, the experiment was conducted on the Ubuntu 20.04 LTS operating system, which offers a stable and efficient Linux environment. For the deep learning framework, PyTorch 1.10.0 was used, a framework with powerful neural network construction and training capabilities, and it is compatible with GPU acceleration to significantly improve training efficiency. The CUDA 11.2 and cuDNN 8.1 libraries were also used to fully leverage the GPU’s computing power and enhance model training speed. For data preprocessing and augmentation, OpenCV 4.5.1 was used, providing efficient image processing functions to meet the needs for operations such as flipping and blurring. To ensure reproducibility, all experiment code was written in Python 3.8 and managed through the Anaconda environment manager to handle the various dependencies.

#### 4.2.2 Hyperparameter settings

In this experiment, the setting of hyperparameters plays a crucial role in the training and performance of the model. The learning rate is set to an initial value of 0.001, with a learning rate decay strategy applied every 10 epochs, reducing the learning rate to 0.1 of its previous value to accelerate the model’s convergence. The batch size is set to 16, balancing memory consumption and training efficiency. The number of training epochs is set to 50, ensuring the model is sufficiently trained. For the optimizer, the Adam optimizer is used with the settings of *β*_1_ = 0.9, *β*_2_ = 0.999, and ϵ = 1*e* − 8, improving the training stability of the model. The loss function is the cross-entropy loss function, suitable for multi-class classification tasks, effectively guiding the model’s training process. To prevent overfitting, L2 regularization (with a weight decay coefficient of 0.0001) and early stopping are applied, where training is stopped early if there is no improvement in the validation loss. Additionally, data augmentation techniques including horizontal flipping, vertical flipping, and blurring are used to enhance the model’s ability to recognize different lesions. By reasonably setting these hyperparameters, the model achieves good performance in plant disease detection tasks.

**Figure 8:**
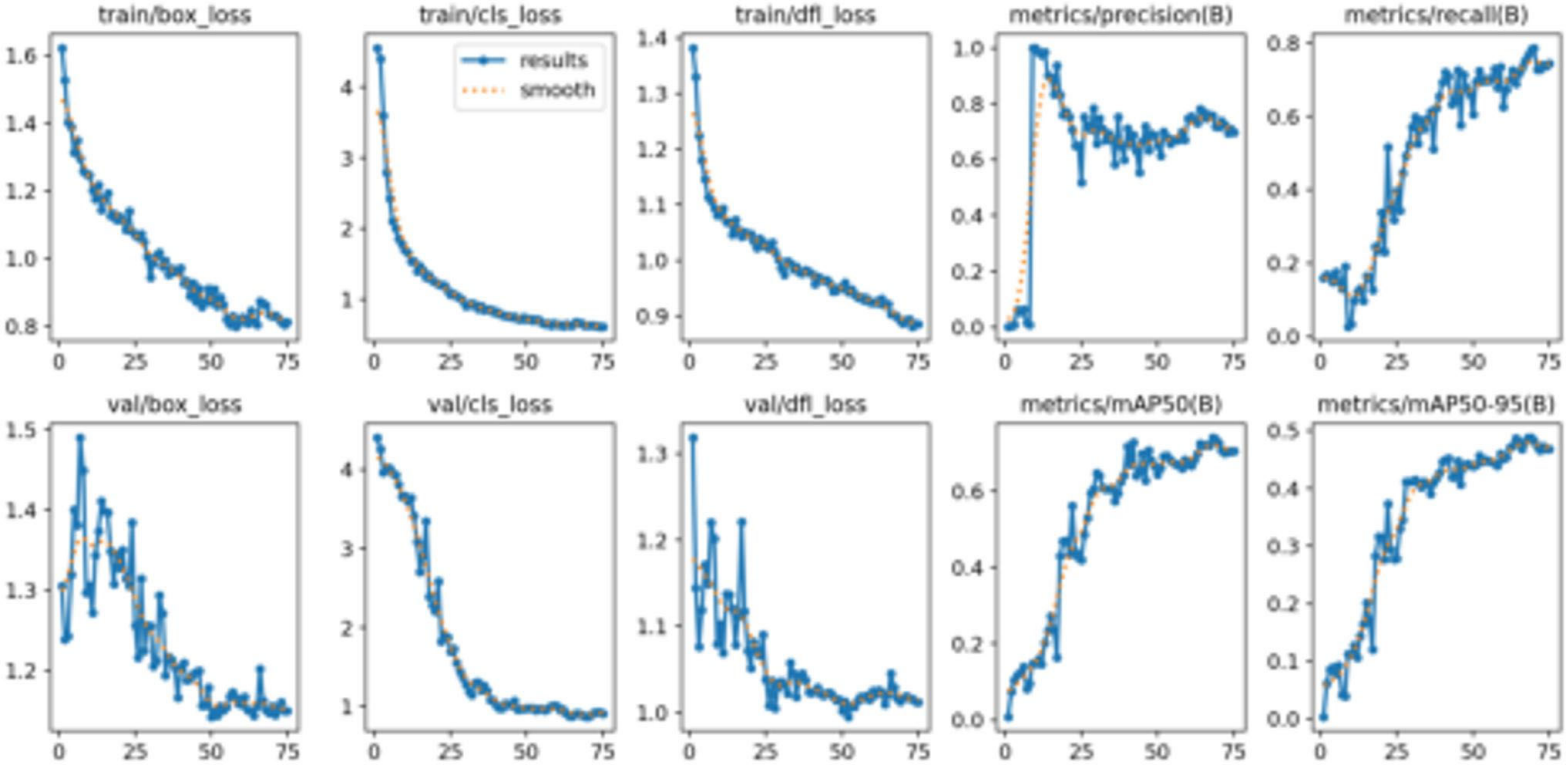
Training and validation results of Plant Pathology 2020 - FGVC7, showing the loss and evaluation metrics (precision, recall, and mAP) over epochs.

The training results of Plant Pathology 2020 - FGVC7 are visualized in **Figure 8**. The graph shows that during training, the loss gradually decreases, and both precision and recall steadily improve, indicating that the model’s performance on the training set is getting better. On the validation set, although the loss fluctuates, the overall trend is decreasing, and the model’s performance continues to improve, particularly in terms of mAP, demonstrating good generalization ability.

#### 4.2.3 Evaluation matrix

In this paper, several commonly used evaluation metrics are employed to assess the performance of the model, including mAP (mean Average Precision), FLOP (Floating Point Operations), and FPS (Frames Per Second). Below is a brief introduction to these evaluation metrics along with their formulas:

mAP is an important metric for evaluating the performance of object detection models, commonly used to assess the average precision at different recall rates. It is calculated by computing the average precision (AP) for each class and then taking the mean of these AP values. The formula is as follows:


$$mAP=\frac{1}{N}\sum_{i=1}^{N}AP_{i}$$

where *N* is the number of classes, and *AP_i_* is the average precision for the *i*-th class.

FLOP is used to measure the computational cost of the model during the inference process, typically assessing the model’s computational complexity. FLOP represents the number of floating-point operations required per inference. A lower FLOP indicates higher computational efficiency. For convolutional neural networks, the calculation of FLOP usually depends on the parameters of each convolutional layer and the size of the input feature maps.

FPS indicates the number of frames processed by the model per second, which is a key metric for real-time performance. Higher FPS means the model can perform inference more quickly, making it suitable for real-time applications. The formula is:


$$FPS=\frac{\text{Total Number of Frames}}{\text{Total Time Taken}}$$

where “Total Number of Frames” represents the total number of frames processed during testing, and “Total Time Taken” represents the total time taken for inference (in seconds).

### 4.3 Comparisons with state-of-the-art detection methods

#### 4.3.1 Plant Pathology 2020 - FGVC7

As shown in **Table 2**, our model has made significant improvements in performance on the Plant Pathology 2020 - FGVC7 dataset compared to several existing mainstream models, particularly in terms of accuracy and computational efficiency. Specifically, the model achieves. 91.0% in mAP@0.5%, which is an improvement of approximately 4.1% over YOLOv11 (86.9%); and 43.0% in mAP@0.5-0.95%, which is an increase of 2.8% over YOLOv11 (40.2%). This demonstrates that our improved model performs significantly better in multi-scale object detection tasks.

**Table 2:** Comparison of model performance on Plant Pathology 2020 - FGVC7.

Models	mAP@0.5	mAP@0.5-0.95	Parameters	Model size/MB	FLOPs/G	FPS
RetinaNet Peng et al. (2022)	69.8%	30.2%	4,570,023	9.1	14.4	128
EfficientDet Tan et al. (2020)	73.2%	33.3%	5,852,863	10.5	13.1	146
M2Det Zhao et al. (2019)	75.0%	33.8%	4,863,463	9.7	12.1	139
SSD Liu et al. (2016)	81.9%	37.3%	4,781,031	9.5	11.9	137
Cascade RCNN Cai and Vasconcelos (2018)	80.0%	28.3%	5,524,356	10.8	11.9	129
Faster RCNN Chen and Gupta (2017)	83.6%	33.6%	4,872,579	9.2	11.9	128
YOLOv5 Chen et al. (2022)	88.2%	39.3%	7,037,095	13.8	15.8	158
YOLOv7 Wang et al. (2023b)	86.0%	30.0%	2,772,695	7.7	6.2	143
YOLOv8 Wang and Liu (2024)	90.6%	37.3%	7,879,630	14.5	14.6	157
YOLOv9 Wang et al. (2024c)	90.2%	40.2%	3,152,334	6.6	8.5	144
YOLOv10 Khanam and Hussain (2024)	85.8%	41.0%	1,125,471	4.4	7.8	176
YOLOv11 Chen et al. (2024)	86.9%	40.2%	1,387,524	4.3	6.4	167
Ours	91.0%	43.0%	1,187,524	4.8	7.3	169

In terms of the number of parameters and model size, our model maintains a relatively compact structure, with 1,187,524 parameters and a model size of 4.8MB. Compared to YOLOv5 (7,037,095 parameters, 13.8MB) and YOLOv8 (7,879,630 parameters, 14.5MB), it is notably lighter. This makes our model more suitable for deployment on resource-constrained devices while maintaining high accuracy, especially in scenarios that require fast responses. Regarding computational cost (FLOPs), our model has 7.3G FLOPs and an inference speed (FPS) of 169. Compared to YOLOv11 (6.4G FLOPs, 167 FPS), this represents an improvement of 0.9G FLOPs and 2 FPS. This optimization allows us to perform real-time detection more efficiently while ensuring high accuracy, meeting the speed requirements in practical applications.

#### 4.3.2 Plant Pathology 2021 - FGVC8

As shown in **Table 3**, our model has achieved significant performance improvements on the Plant Pathology 2021 - FGVC8 dataset. Specifically, the model achieves 94.0% in mAP@0.5%, which is a 5.1% improvement over YOLOv11 (88.9%); and 46.0% in mAP@0.5-0.95%, an increase of 6.8% compared to YOLOv11 (39.2%). This indicates that our model performs more accurately in agricultural disease detection tasks, especially in detecting small and multi-scale targets in complex backgrounds. Despite having a compact structure with 1,194,478 parameters and a model size of 4.8MB, our model still maintains high accuracy, being lighter than YOLOv5 (7,037,095 parameters, 13.8MB) and YOLOv8 (7,879,630 parameters, 14.5MB), making it well-suited for deployment on resource-constrained devices. Additionally, the model has 7.3G FLOPs and an inference speed (FPS) of 169. Although the inference speed is slightly lower than YOLOv11 (179 FPS), the substantial increase in detection accuracy still allows for real-time monitoring tasks. Overall, our model demonstrates excellent performance in both accuracy and computational efficiency, with significant progress in small target detection and multi-scale feature fusion, showcasing its strong potential in practical applications such as agricultural disease detection.

**Table 3:** Comparison of model performance on Plant Pathology 2021 – FG VC8.

Models	mAP@0.5	mAP@0.5-0.95	Parameters	Model size/MB	FLOPs/G	FPS
RetinaNet Peng et al. (2022)	79.9%	37.2%	4,570,023	9.1	14.4	140
EfficientDet Tan et al. (2020)	77.1%	39.3%	5,852,863	10.5	13.1	146
M2Det Zhao et al. (2019)	84.9%	38.8%	4,863,463	9.7	12.1	139
SSD Liu et al. (2016)	87.2%	36.3%	4,781,031	9.5	11.9	137
Cascade RCNN Cai and Vasconcelos (2018)	90.1%	38.3%	5,524,356	10.8	11.9	158
Faster RCNN Chen and Gupta (2017)	83.6%	39.6%	4,872,579	9.2	11.9	139
YOLOv5 Chen et al. (2022)	91.2%	41.1%	7,037,095	13.8	15.8	178
YOLOv7 Wang et al. (2023b)	86.9%	39.0%	2,772,695	7.7	6.2	153
YOLOv8 Wang and Liu (2024)	86.3%	42.3%	7,879,630	14.5	14.6	167
YOLOv9 Wang et al. (2024c)	92.2%	39.2%	3,152,334	6.6	8.5	145
YOLOv10 Wang et al. (2024a)	89.8%	40.7%	1,125,471	4.4	7.8	186
YOLOv11 Khanam and Hussain (2024)	88.9%	39.2%	1,387,524	4.3	6.4	179
Ours	94.0%	46.0%	1194,478	4.8	7.3	169

### 4.4 Ablation experiment

As shown in **Table 4**, we conducted ablation experiments to analyze the contribution of each module to the model’s performance. The experimental results demonstrate that the progressive introduction of the CSPPA, SEA, and LGCK modules significantly improves the model’s performance in agricultural disease detection tasks. First, the CSPPA module enhances multi-scale information fusion, improving the model’s ability to detect targets of various scales, especially small targets in complex backgrounds. Next, the SEA module, by integrating contextual and spatial features, strengthens the model’s ability to capture both global and local details, further improving detection accuracy across multiple scales. The LGCK module optimizes the detection of small targets by refining feature extraction, increasing the model’s sensitivity to fine details. Ultimately, the combination of all three modules achieves the best performance, fully leveraging the strengths of each, particularly in small target detection and multi-scale feature fusion, leading to significant improvements in overall accuracy. These results align with our initial design approach, confirming the effectiveness of CSPPA, SEA, and LGCK in enhancing agricultural disease detection, especially in the precise recognition of multi-feature, multi-scale targets in complex backgrounds.

**Table 4:** Experiment results for each component.

Method	CSPPA	SEA	LGCK	mAP@0.5	mAP@0.5-0.95
YOLOv11	✘	✘	✘	0.869	0.402
YOLOv11-1	**√**	✘	✘	0.871	0.418
YOLOv11-2	✘	**√**	✘	0.882	0.419
YOLOv11-3	✘	✘	**√**	0.902	0.423
YOLOv11-4	**√**	**√**	✘	0.905	0.425
YOLOv11-5	✘	**√**	**√**	0.908	0.421
Ours	**√**	**√**	**√**	0.910	0.430

**Figure 9:**
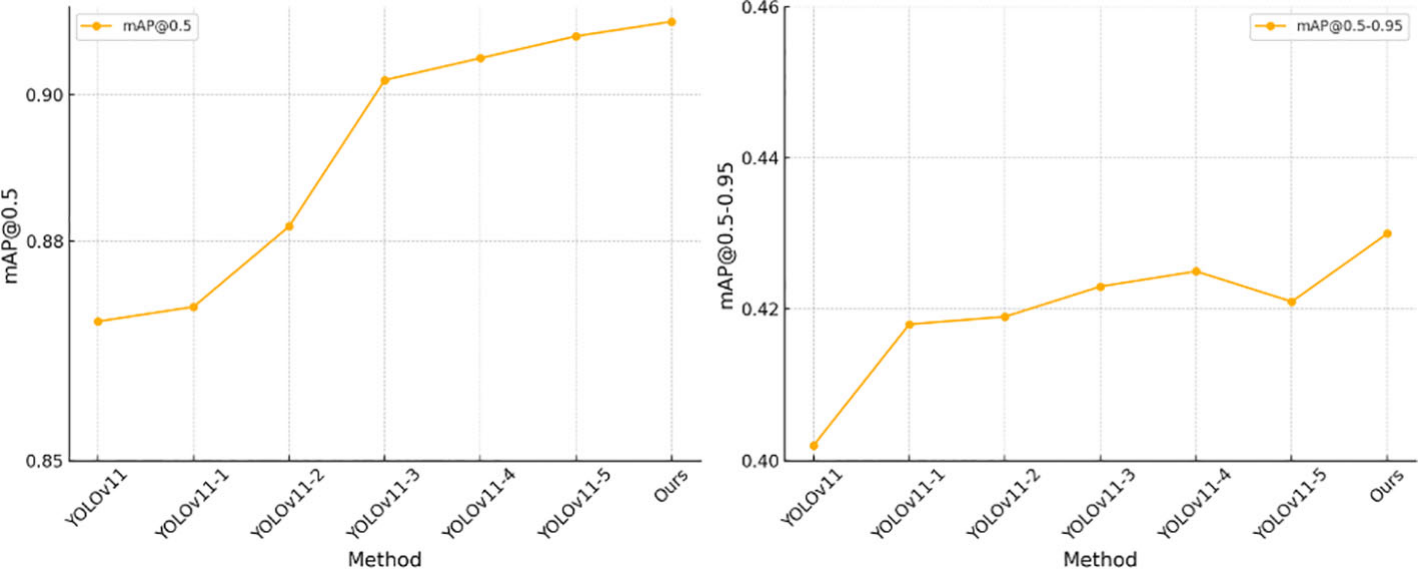
Ablation experiment results display.

**Figure 9** provides a visual representation of the table contents, making it more intuitive to observe the significant improvement achieved by the combination of the three modules.

### 4.5 Qualitative results

**Figure 10:**
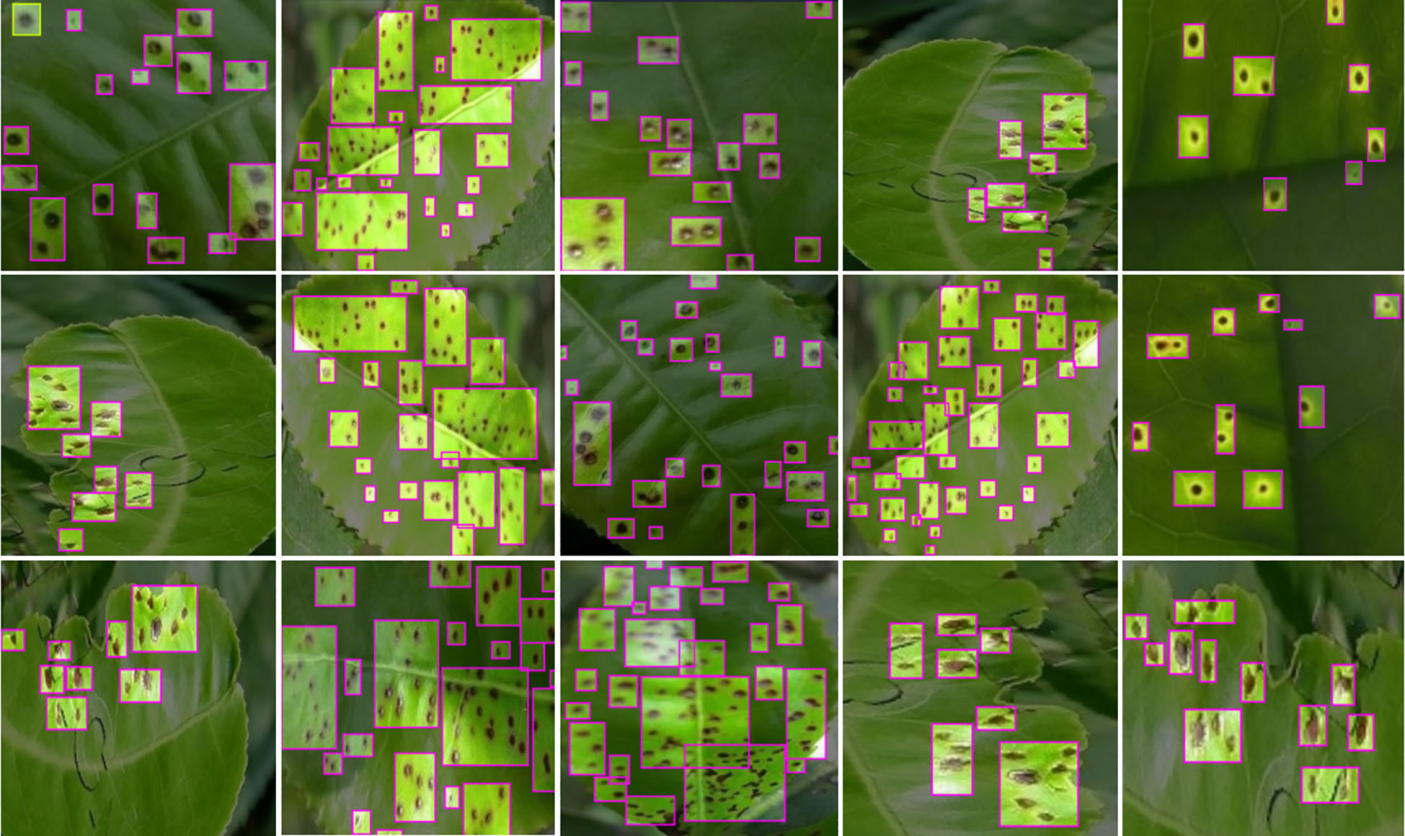
YOLO-LF model’s qualitative results for small target detection on the plant leaf disease dataset. The model demonstrates accurate localization of small lesions, even in complex backgrounds.

As shown in **Figure 10**, we present the qualitative results of small target detection for scab disease on the plant leaf disease dataset. It is evident that our proposed model demonstrates significant advantages in small target localization and identification. Especially in complex backgrounds, the model can accurately identify the diseased areas, maintaining high detection accuracy even in low-contrast and noisy conditions.

For some small lesions, the model is able to clearly mark their positions, whereas other models often suffer from missed detections or false positives.

**Figure 11:**
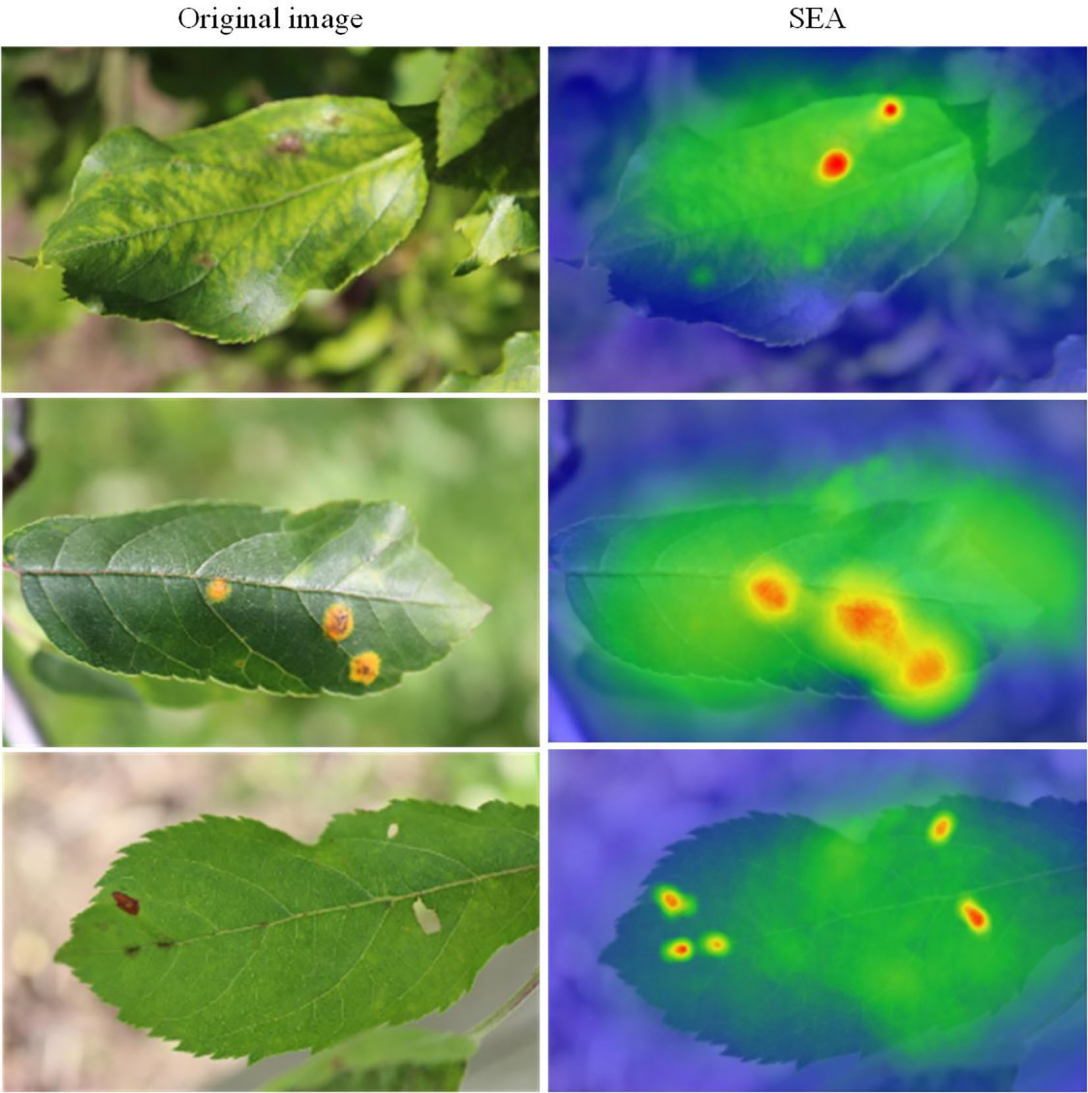
Visualization of SEA attention mechanism.

In different areas of the leaf, especially where the diseased regions are small and widely scattered, our model demonstrates stronger detail-capturing capabilities. Compared to other common object detection models, our model shows better robustness in handling occluded areas, effectively distinguishing diseased regions from the background, thereby significantly reducing false positives. Additionally, we visualized the SEA module using a heatmap (as shown in **Figure 11**), which clearly demonstrates the model’s ability to accurately focus on the diseased areas, while also validating the effectiveness of the CSPPA module. Our model provides more accurate detection results in complex backgrounds and for small targets, which is of great significance for real-time monitoring and accurate diagnosis of agricultural diseases. These improvements indicate that our proposed approach can significantly enhance the detection capability of small target diseases in the agricultural field.

### 4.6 Limitations and future work

Although the model proposed in this paper achieves significant performance improvements in plant disease detection tasks, there are still some limitations. Firstly, our model relies on existing plant leaf disease datasets, which have a relatively limited number of samples and diversity. In particular, there may be data imbalances for certain disease types, which could result in suboptimal performance for specific disease categories or rare lesions. Additionally, although our model enhances small target detection capabilities by incorporating multiple modules, the model’s parameter size and computational load are still relatively large, which may pose deployment challenges on resource-constrained devices. Therefore, further optimization of the model’s computational efficiency and reduction of the parameter size will help improve the model’s usability in practical applications.

Future research could focus on addressing these limitations. First, by expanding plant disease datasets, especially those covering a wider variety of disease types and diverse backgrounds, we can enhance the model’s generalization ability. Additionally, exploring optimization methods based on lightweight network architectures to reduce the model’s parameter size and computational cost would improve its deployment efficiency. Another direction could be introducing self-supervised learning or reinforcement learning techniques to further improve the model’s performance in data-scarce or imbalanced scenarios. Furthermore, combining data from other sensors (such as images, temperature, humidity, etc.) for multimodal fusion may provide richer information to enhance the model’s robustness and accuracy in complex environments.

## 5 Conclusion

This paper proposes an improved YOLO-LF model aimed at enhancing the detection accuracy of small targets in agricultural disease detection. By introducing modules such as CSPPA, SEA, and LGCK, the model effectively integrates multi-scale features and detail information, demonstrating significant advantages in detecting small lesions in complex backgrounds. The CSPPA module enhances multiscale information fusion, the SEA module improves the ability to capture contextual and spatial details, and the LGCK module plays a key role in small target detection. Experimental results show that the improved model not only achieves significant improvements in detection accuracy compared to existing mainstream models, especially in mAP@0.5% and mAP@0.5-0.95%, but also achieves a good balance between computational efficiency and inference speed. Future research could focus on further enhancing the model’s generalization ability and efficiency by expanding the dataset, introducing lightweight network architectures, and integrating more sensor data. With these optimizations, we believe the model’s application potential in agricultural disease monitoring will be even broader, particularly for real-time monitoring and precise diagnosis in complex environments. Overall, this study demonstrates the immense potential of the YOLO-LF model in agricultural disease detection tasks, providing an effective solution with high practical application value.

## Data Availability

The original contributions presented in the study are included in the article/supplementary material. Further inquiries can be directed to the corresponding authors.
